# Factors Related to Lacking Knowledge on the Recommended Daily Salt Intake among Medical Professionals in Mongolia: A Cross-Sectional Study

**DOI:** 10.3390/ijerph18083850

**Published:** 2021-04-07

**Authors:** Naoko Hikita, Enkhtungalag Batsaikhan, Satoshi Sasaki, Megumi Haruna, Ariunaa Yura, Otgontogoo Oidovsuren

**Affiliations:** 1 Graduate Program of Midwifery, Dokkyo Medical University, 880 Kitakobayashi, Mibu, Shimotsuga-gun, Tochigi 321-0293, Japan; 2 Department of Midwifery and Women’s Health, Division of Health Sciences and Nursing, Graduate School of Medicine, The University of Tokyo, Tokyo 113-0033, Japan; mharuna@m.u-tokyo.ac.jp; 3 Department of Nutrition Research of the National Center for Public Health, Ulaanbaatar 210349, Mongolia; tunga25@ymail.com; 4 Department of Social and Preventive Epidemiology, School of Public Health, Graduate School of Medicine, The University of Tokyo, Tokyo 113-0033, Japan; stssasak@m.u-tokyo.ac.jp; 5 Darkhan-Uul General Hospital, Darkhan-Uul 45051, Mongolia; ariunaa_0807@yahoo.com; 6 Darkhan-Uul Medical School, Mongolian National University of Medical Sciences, Darkhan-Uul 45051, Mongolia; otgontogoo@mnums.edu.mn

**Keywords:** medical professionals, knowledge, practices, salt intake, Mongolia

## Abstract

In Mongolia, the recommendations are to restrict salt intake to less than 5 g/day to reduce the risk of cardiovascular disease. We aimed to reveal factors associated with not knowing the recommended daily salt intake among medical professionals in Mongolia. Of the recruited 538 medical professionals working at public health facilities in Darkhan-Uul Province, the data from 338 (62.8%), obtained using self-administered questionnaires, were analyzed. Among these, 175 (51.8%) did not know the recommended daily salt intake. Compared with medical doctors, midwives and nurses had higher odds of not knowing the recommendations (adjusted odds ratio (AOR): 4.20, 95% confidence interval (CI): 1.40–12.59; AOR: 2.10, 95% CI: 1.15–3.76, respectively). Compared to participants who consumed more than four cups/day of salted *suutei tsai* (Mongolian milk tea), those who consumed approximately two cups/week had lower odds of not knowing the recommendations (AOR: 0.21, 95% CI: 0.07–0.63). With most participants lacking accurate knowledge on this topic, and considering that people who are aware of the recommendations are more likely to take action to reduce dietary salt intake, it is imperative to urgently address this knowledge deficit because medical professionals have a responsibility to educate the community by disseminating accurate health information.

## 1. Introduction

Sodium is an essential mineral for humans, with most of the sodium that we consume being in the form of salt. Sodium is naturally present in a variety of foods, such as meat and fish, and processed foods (breads, processed meats, and snack foods) often contain excessive amounts of sodium [1,2,3]. Many studies report that excessive sodium intake can cause several diseases, such as cardiovascular diseases, hypertension, and stomach cancer [4,5,6,7,8,9]. Therefore, the World Health Organization (WHO) recommends that adults consume less than 2 g/day of sodium (equivalent to less than 5 g/day of salt) [3]. This recommendation has been adopted by several countries, including Mongolia.

Previous studies reported that the average daily salt intake in Mongolia exceeded 10 g/day, which is more than double the recommended amount [10,11]. Excessive salt intake might affect cardiovascular disease, which is the leading cause of death in Mongolia [12]. The Ministry of Health therefore supports the recommendations of the WHO: adults should restrict their salt intake to less than 5 g/day [11]. Medical professionals play an essential role in disseminating health information to the community, especially medical professionals who work in public health facilities. Thus, they must have accurate knowledge of the current health guidelines, including salt intake recommendations, because having knowledge regarding salt is inversely related to the use of salt [13]. However, there are no reports that have evaluated the knowledge of medical professionals in Mongolia regarding the recommended daily salt intake for adults, or the factors associated with lacking knowledge regarding this recommendation. Therefore, the aim of this study was (1) to investigate the knowledge of the recommended daily salt intake for adults and (2) to reveal the factors associated with lacking knowledge on the recommended daily salt intake among medical professionals in Darkhan-Uul Province, Mongolia.

## 2. Materials and Methods

### 2.1. Study Design and Population

This cross-sectional study was part of a broader research study that investigated sodium excretion using 24-h urine collection; this paper focuses only on medical professionals’ knowledge and practices regarding the recommended salt intake level. The full study was conducted from September to October 2019 in Darkhan-Uul Province, Mongolia. All medical doctors, associate medical doctors, midwives, nurses, and public health nurses who worked in public health facilities in Darkhan-Uul Province were included in the recruitment process. There were no exclusion criteria. There were 538 medical professionals (58 male and 480 female) in Darkhan-Uul Province at the time, from 10 public health facilities, including the Health Department, one general hospital, three *soum* (village) hospitals, and five health centers. Before starting the study, approval was obtained from the Director of the Health Department. The head of each health facility was informed about the objectives of the study and the schedule, and permission was granted by each before the study commenced.

### 2.2. Data Collection

Before starting the recruitment process, eight research assistants took part in a one-day training session. The purpose of the study was explained, as well as the methods of the investigation and how consent should be obtained from the participants. The trained research assistants visited each health facility, recruited medical professionals, and collected the data reported in this paper using self-administered questionnaires. Participants were informed that they would be provided with the results of their estimated salt intake and sodium excretion if they participated in the study.

Participants answered questions relating to their age, sex, occupation (medical doctor, associate medical doctor, midwife, nurse, or public health nurse), and knowledge of the recommended salt intake. Participants were asked “Do you know the recommended amount of salt intake in Mongolia?” to confirm if they had knowledge about recommended salt intakes. If they answered “Yes,” they were asked to report the recommended amount of salt intake per day (g/day). If they answered “5 g/day,” they were considered to have knowledge of the recommended daily salt intake, whereas if they did not answer correctly they were considered to lack the relevant knowledge. Furthermore, participants were asked to report on their practices related to salt intake, such as how often they added salt or sauce to their food at the table (always, often, sometimes, rarely, or never), ate processed foods that are high in salt (4 times/day or more, 3 times/day, 2 times/day, once a day, once/2–3 days, or once/4–6 days), drank salted *suutei tsai* (Mongolian milk tea) (4 cups/day or more, about 2 cups/day, about 1 cup/day, 1 cup/2–3 days, 1 cup/4–6 days, 1 cup/week or less, or not drink), as well as whether they read the information on salt or sodium on food labels (always, sometimes, or never). “How much soup do you eat when you eat soup dishes?” (almost none, about 20%, about 40–60%, about 80%, or almost all) was also asked because a previous study reported that the proportion of consumed noodle soup was associated with higher sodium excretion [14]. Information on whether they had taken oral antihypertensive medicine or a diuretic within the past two weeks (yes or no), or if they had been diagnosed with renal disease (yes or no), was also collected.

Body height, weight, and blood pressure were measured by research assistants using a digital body composition weighing scale (BC-758, TANITA Corp., Tokyo, Japan), a stadiometer, and a digital sphygmomanometer (ES-H56, TERUMO Corp., Tokyo, Japan). The body mass index (BMI) was calculated using body weight and height: BMI = weight (kg)/height (m)^2^.

### 2.3. Statistical Analysis

Descriptive statistics were used to show the demographics of participants and their practices related to salt intake. Categorical data are presented as n (%), whereas continuous variables are presented as mean ± standard deviation. We used the chi-square test for categorical data and Student’s *t*-test for the comparison of continuous variables between the groups with and without knowledge of the recommended salt intake. The variables that showed a tendency towards a difference between the groups with and without knowledge (*p* < 0.1) were selected as independent variables for multiple logistic regression analysis. Multicollinearity was evaluated using Spearman’s rank correlation coefficient or Cramer’s V; if the correlation coefficient exceeded 0.5, one of the variables was removed from the multiple logistic regression analysis. All data were analyzed using IBM SPSS Statistics 25.0 for Windows (IBM Corp., Armonk, NY, USA). All two-tailed *p*-values < 0.05 were considered statistically significant.

## 3. Results

### 3.1. Characteristics of the Study Participants

Of the 538 medical professionals in the province, 352 agreed to participate; however, 14 did not fulfill all the requirements for participation. Thus, the final dataset included 338 (62.8%) participants.

Table 1 shows the characteristics of the participants. The participants’ average age was 40.3 ± 11.1 years, and 320 (94.7%) were female. Regarding their occupation, 91 (26.9%) were medical doctors and 176 (52.1%) were nurses. Among the 338 participants, 163 (48.2%) knew the recommended daily amount of salt intake, and 175 (51.8%) did not. The participants’ average BMI was 26.6 ± 4.4 kg/m^2^, systolic blood pressure was 115.0 ± 15.0 mm Hg, and diastolic blood pressure was 73.2 ± 10.9 mm Hg. The age group and occupation of the participants differed between the group with and without knowledge (*p* < 0.1).

### 3.2. Practices Related to Salt Intake

Table 2 shows the participants’ practices related to salt intake. More than 80% of the participants (*n* = 281, 83.6%) used iodized salt, 86 (25.5%) never added salt or sauce to food while cooking, 108 (32.0%) never added salt or sauce to their food at the table, 139 (41.2%) never paid attention to the salt content specified on food labels, and 137 (40.8%) never used spices during cooking. A difference was found between the group with and without knowledge (*p* < 0.1) regarding how often salt or sauce was added to food at the table and during cooking or preparation of meals, the amount of salted *suutei tsai* consumed, and the amount of soup consumed when eating soup dishes.

### 3.3. Factors Related to Lacking Knowledge on the Recommended Salt Intake

Table 3 shows the results of multiple logistic regression analysis for factors related to lacking knowledge on the recommended salt intake. Compared to participants aged 20–29 years, those aged 30–39 years had lower odds of lacking knowledge (adjusted odds ratio (AOR): 0.38, 95% confidence interval (CI): 0.19–0.77). Moreover, compared to medical doctors, midwives and nurses had higher odds of lacking knowledge on the recommended salt intake (AOR: 4.20, 95% CI: 1.40–12.59; AOR: 2.10, 95% CI: 1.15–3.76, respectively). Compared to participants who drank more than four cups/day of salted *suutei tsai*, those who drank approximately two cups/week had lower odds of not knowing the recommended salt intake (AOR: 0.21, 95% CI: 0.07–0.63).

## 4. Discussion

To our knowledge, this is the first study to reveal the factors related to Mongolian medical professionals lacking knowledge on the recommended salt intake. Our results show that 51.8% of medical professionals lacked this knowledge. Participants’ age, occupation, certain practices related to salt intake—such as how often they added salt or sauce to food while cooking and the amount of salted *suutei tsai* consumed—significantly differed between the groups with and without knowledge.

### 4.1. Factors Related to Lacking Knowledge on the Recommended Salt Intake

In this study, more than half of the participants lacked knowledge on the recommended daily salt intake despite all being medical professionals. In Mongolia, medical professionals are responsible for educating the community about their health, thus all medical professionals must know the recommendations for daily salt intake (5 g/day). A previous study investigated the role and skills of nurses providing hypertension care; it was found that educating patients on changing lifestyle behaviors, including salt restriction, was the nurse’s role [15]. However, our results show that midwives and nurses were significantly more likely to lack knowledge on the recommended daily salt intake than medical doctors were, although significant differences were not found between medical doctors and public health nurses. This finding is similar to that from a previous study that revealed health care professionals’ knowledge regarding alcohol consumption differed significantly depending on the type of occupation [16]. The reason significant differences were not found between medical doctors and public health nurses might be due to the small sample size of public health nurses or because the role of public health nurses is to give health guidance to the community; in addition, some of the nurses were not specialists in public health or cardiovascular internal medicine. In Mongolia, based on the number per 1000 population, nurses make up the largest cadre of health professionals [17], thus, improving the level of nurses’ public health knowledge is essential and would be an effective way to disseminate accurate information on the importance of salt reduction to the community. Identifying knowledge gaps among medical professionals may help to determine focus areas for continuing medical education for health professionals. Furthermore, well-informed medical professionals may help to improve the dissemination of public health information in Mongolia.

In this study, compared to participants aged 20–29 years, those aged 30–39 were significantly less likely to lack knowledge on the recommended daily salt intake. This finding is similar to that of a previous study that reported older people to have superior knowledge regarding the harmful effect of salt on health [18], but is different to that from another previous study that found age to be unrelated to the level of knowledge of salt intake among nonmedical professionals in African countries [19]. It was expected that the knowledge of medical professionals would increase with age, and a relationship between these variables was found in this study. However, compared to participants aged 20–29 years, this relationship was not found for those participants aged 40 years or older. The reason why this relationship was not found might be that the concept of excess salt consumption being a major risk factor for several diseases is still relatively new in Mongolia; thus, it was not proportional to participant age, which is a proxy for years of clinical experience.

Regarding how often salt or sauce was added to food during cooking or preparation of meals, participants who rarely added salt or sauce were more likely to lack knowledge on the recommended daily salt intake as compared to those who never added salt or sauce. This may indicate that participants who knew the recommended daily salt intake paid more attention to the amount of salt they added to food. A previous study reported that people who are aware of the daily limit of salt intake are more likely to take action to reduce dietary salt consumption [20]; thus, our results supported the results of this previous study.

Compared to participants who consumed more than four cups/day of salted *suutei tsai*, those who consumed approximately two cups/day and those who consumed one cup every 4–6 days were less likely to lack knowledge on the recommended daily salt intake. This finding also supports those from a previous study reporting that people who are aware of the daily limit of salt intake are more likely to take action to reduce dietary salt consumption [20]. In Mongolia, people who consume salted *suutei tsai* consume significantly more salt on average [21]; thus, a focus on salted *suutei tsai* consumption may be important to reduce salt intake in this population, also because salt-related practices are negatively associated with sodium intake [22].

### 4.2. Strengths and limitations

The main strength of this study is that it was the first study to reveal factors associated with lacking knowledge on salt intake recommendations among medical professionals who work at public health facilities in Mongolia. As medical professionals have the responsibility to disseminate health education to the community, it is essential for them to have accurate knowledge about salt intake recommendations. Furthermore, all medical professionals who worked at public health facilities across the province were included in this study, and some questions used in this study were the same questions as those in the STEPS survey in Mongolia [23]; thus, the results of this study may be relevant for investigating the knowledge of health professionals in other provinces as well.

Despite several strengths, this study also has several limitations. First, this study adopted a cross-sectional design; therefore, it cannot be used to infer a causal relationship between knowledge and practices related to salt intake. Second, we had no information about participants’ department or their specialties; thus, we did not consider these factors in the analysis. Some health professionals may not require knowledge on the recommended salt intake because it is not their specialty or service area. Third, the participation ratio of male medical professionals (31.0%, 18/58) was lower than that of female medical professionals (66.7%, 211/320), which may have biased the results. Fourth, we evaluated the knowledge of salt intake based on only one question regarding 5 g/day as the recommended amount of daily salt intake. Moreover, the questionnaire we used was original. A further study that uses a validated questionnaire or assessment tool is needed to examine knowledge about salt intake. Fifth, there were many participants who took oral antihypertensive medicine and who had been diagnosed with renal disease. This might have affected their knowledge and practices related to salt intake. Different results might be obtained with a different sample of participants.

## 5. Conclusions

This study revealed factors associated with lacking knowledge on the recommended daily salt intake for adults among medical professionals who work at public health facilities in Darkhan-Uul Province, Mongolia. Participants’ age, occupation, and certain practices related to salt intake were significantly associated with lacking knowledge on the recommended daily salt intake. Although medical professionals have the important role of providing health education to the community, most did not possess accurate knowledge regarding the recommendations for daily salt intake. Medical professionals’ deficits in knowledge must be addressed, especially considering that they are required to provide health education to the public.

## Figures and Tables

**Table 1:** Characteristics of participants (*n* = 338).

Characteristics	All		Without Knowledge		With Knowledge		*p*
	(*n* = 338)		(*n* = 175, 51.8%)		(*n* = 163, 48.2%)		
	Mean ± SD or n (%)		Mean ± SD or n (%)		Mean ± SD or n (%)		
Age (years)	40.3 ± 11.1		39.4 ± 11.1		41.2 ± 11.1		0.121
Age group (years)							0.052
20–29	81	(24.0)	51	(63.0)	30	(37.0)	
30–39	84	(24.8)	34	(40.5)	50	(59.5)	
40–49	79	(23.4)	43	(54.4)	36	(45.6)	
50–59	88	(26.0)	45	(51.1)	43	(48.9)	
60+	6	(1.8)	2	(33.3)	4	(66.7)	
Sex							0.194
Male	18	(5.3)	12	(66.7)	6	(33.3)	
Female	320	(94.7)	163	(50.9)	157	(49.1)	
Occupation							0.010
Medical doctor	91	(26.9)	36	(39.6)	55	(60.4)	
Associate medical doctor	38	(11.2)	20	(52.6)	18	(47.4)	
Midwife	25	(7.4)	18	(72.0)	7	(28.0)	
Nurse	176	(52.1)	99	(56.3)	77	(43.7)	
Public health nurse	8	(2.4)	2	(25.0)	6	(75.0)	
Body height (cm) (*n* = 336)	159.2 ± 6.5		159.5 ± 6.6 ^a^		158.9 ± 6.4 ^a^		0.365 ^b^
Body weight (kg)	67.6 ± 12.2		67.3 ± 10.8		67.9 ± 13.6		0.621 ^b^
Body mass index (*n* = 336)	26.6 ± 4.4		26.4 ± 3.8 ^a^		26.9 ± 4.9 ^a^		0.294 ^b^
Systolic blood pressure (mm Hg) (*n* = 337)	115.0 ± 15.0		115.2 ± 14.7		114.7 ± 15.4 ^a^		0.777 ^b^
Diastolic blood pressure (mm Hg) (*n* = 337)	73.2 ± 10.9		73.0 ± 11.0		73.4 ± 10.8 ^a^		0.682 ^b^
Taking oral antihypertensive medicine							0.553
Yes	64	(18.9)	31	(48.4)	33	(51.6)	
No	274	(81.1)	144	(52.6)	130	(47.4)	
Taking oral diuretic (*n* = 337)							0.466
Yes	13	(3.9)	8	(61.5)	5	(38.5)	
No	324	(96.1)	166	(51.2)	158	(48.8)	
Having diagnosis of renal disease							0.194
Yes	129	(38.2)	61	(47.3)	68	(52.7)	
No	209	(61.8)	114	(54.5)	95	(45.5)	

Chi-square test, SD: standard deviation, ^a^ 1 data point was missing, ^b^ Student’s *t*-test.

**Table 2:** Participants’ practices related to salt intake (*n* = 338).

Practices Related to Salt Intake	All		Without Knowledge		With Knowledge ^a^		*p*
	(*n* = 338)		(*n* = 175, 51.8%)		(*n* = 163, 48.2%)		
	n (%)		n (%)		n (%)		
Using iodized salt (*n* = 336)							0.662
Yes	281	(83.6)	147	(52.3)	134	(47.7)	
No	55	(16.4)	27	(49.1)	28	(50.9)	
Buying low salt/sodium alternatives (*n* = 334)							
Always	19	(5.7)	8	(42.1)	11	(57.9)	0.568
Sometimes	185	(55.4)	95	(51.4)	90	(48.6)	
Never	130	(38.9)	71	(54.6)	59	(45.4)	
Frequency of adding salt or sauce to food at the table (*n* = 337)							
Always	10	(3.0)	8	(80.0)	2	(20.0)	0.004
Sometimes (often, rarely)	219	(65.0)	123	(56.2)	96	(43.8)	
Never	108	(32.0)	43	(39.8)	65	(60.2)	
Amount of salt or sauce usage when adding at the table (*n* = 310)							0.178
A little	264	(85.2)	136	(51.5)	128	(48.5)	
Medium	41	(13.2)	26	(63.4)	15	(36.6)	
A lot	5	(1.6)	4	(80.0)	1	(20.0)	
Frequency of adding salt or sauce to food while cooking (*n* = 337)							0.032
Always	10	(3.0)	5	(50.0)	5	(50.0)	
Sometimes (often, rarely)	241	(71.5)	135	(56.0)	106	(44.0)	
Never	86	(25.5)	34	(39.5)	52	(60.5)	
Frequency of eating processed foods high in salt (ham, salami, cheese, pickles, bread, ramen, etc.) (*n* = 306)							0.694
4 times/day or more	3	(1.0)	2	(66.7)	1	(33.3)	
3 times/day	7	(2.3)	5	(71.4)	2	(28.6)	
2 times/day	24	(7.8)	10	(41.7)	14	(58.3)	
Once a day	49	(16.0)	25	(51.0)	24	(49.0)	
Once/2–3 days	50	(16.3)	29	(58.0)	21	(42.0)	
Once/4–6 days	173	(56.5)	90	(52.0)	83	(48.0)	
Reading the information on salt or sodium on food labels (*n* = 337)							0.287
Always	33	(9.8)	13	(39.4)	20	(60.6)	
Sometimes	165	(49.0)	85	(51.5)	80	(48.5)	
Never	139	(41.2)	76	(54.7)	63	(45.3)	
Using spices other than salt during cooking (*n* = 336)							0.777
Always	19	(5.7)	11	(57.9)	8	(42.1)	
Sometimes	180	(53.6)	91	(50.6)	89	(49.4)	
Never	137	(40.8)	73	(53.3)	64	(46.7)	
Amount of salted *suutei tsai* consumed (*n* = 337)							0.014
4 cups/day or more	68	(20.2)	43	(63.2)	25	(36.8)	
About 2 cups/day	86	(25.5)	41	(47.7)	45	(52.3)	
About 1 cup/day	42	(12.5)	20	(47.6)	22	(52.4)	
1 cup/2–3 days (About 3 cups/week)	34	(10.1)	23	(67.6)	11	(32.4)	
1 cup/4–6 days (About 2 cups/week)	25	(7.4)	6	(24.0)	19	(76.0)	
1 cup/week or less	56	(16.6)	29	(51.8)	27	(48.2)	
None	26	(7.7)	12	(46.2)	14	(53.8)	
Frequency of soup dishes intake (*n* = 337)							0.676
4 times or more/day	30	(8.9)	19	(63.3)	11	(36.7)	
About 2 times/day	48	(14.2)	22	(45.8)	26	(54.2)	
About once a day	130	(38.6)	67	(51.5)	63	(48.5)	
Once/2–3 days	107	(31.8)	56	(52.3)	51	(47.7)	
Once/4–6 days	22	(6.5)	11	(50.0)	11	(50.0)	
Amount of soup consumed when eating soup dishes (*n* = 337)							0.099
Almost none	5	(1.5)	3	(60.0)	2	(40.0)	
About 20%	28	(8.3)	20	(71.4)	8	(28.6)	
About 40–60%	71	(21.1)	41	(57.7)	30	(42.3)	
About 80%	39	(11.6)	21	(53.8)	18	(46.2)	
Almost all	194	(57.6)	90	(46.4)	104	(53.6)	

Chi-square test. ^a^ Participants were considered to have knowledge if they answered “5 g/day” as the recommended amount of daily salt intake.

**Table 3:** Factors related to lacking knowledge on the recommended daily salt intake.

Variables	Crude Odds Ratio	95% CI	*p*	Adjusted Odds Ratio	95% CI	*p*
Age group (years)						
20–29	Reference			Reference		
30–39	0.40	(0.21–0.75)	0.004	0.38	(0.19–0.77)	0.007
40–49	0.70	(0.37–1.32)	0.274	0.74	(0.36–1.51)	0.404
50–59	0.62	(0.33–1.14)	0.122	0.66	(0.33–1.31)	0.233
60+	0.29	(0.05–1.70)	0.172	0.27	(0.04–1.93)	0.191
Occupation						
Medical doctor	Reference			Reference		
Associate medical doctor	1.70	(0.79–3.64)	0.174	1.71	(0.72–4.03)	0.224
Midwife	3.93	(1.49–10.35)	0.006	4.20	(1.40–12.59)	0.010
Nurse	1.96	(1.17–3.29)	0.010	2.10	(1.15–3.76)	0.015
Public health nurse	0.51	(0.10–2.66)	0.424	0.40	(0.07–2.38)	0.316
Frequency of adding salt or sauce to food at the table						
Always	6.05	(1.23–29.85)	0.027	2.78	(0.45–17.15)	0.271
Sometimes (often, rarely)	1.94	(1.21–3.10)	0.006	1.46	(0.79–2.71)	0.229
Never	Reference			Reference		0.283
Frequency of adding salt or sauce to food while cooking						
Always	1.53	(0.41–5.68)	0.526	0.96	(0.19–4.85)	0.957
Sometimes (often, rarely)	1.95	(1.18–3.22)	0.009	1.82	(0.94–3.52)	0.076
Never	Reference			Reference		
Amount of salted *suutei tsai* intake						
4 cups or more/day	Reference			Reference		
About 2 cups/day	0.53	(0.28–1.01)	0.055	0.46	(0.22–0.95)	0.035
About 1 cup/day	0.53	(0.24–1.15)	0.109	0.53	(0.22–1.24)	0.143
1 cup/2–3 days (About 3 cups/week)	1.22	(0.51–2.91)	0.660	0.97	(0.37–2.52)	0.943
1 cup/4–6 days (About 2 cups/week)	0.18	(0.07–0.52)	0.001	0.21	(0.07–0.63)	0.005
1 cup/week or less	0.62	(0.30–1.28)	0.200	0.56	(0.26–1.22)	0.146
None	0.50	(0.20–1.24)	0.136	0.80	(0.30–2.15)	0.653
Amount of soup consumed when eating soup dishes						
Almost none	Reference			Reference		
About 20%	1.67	(0.23–11.93)	0.611	2.54	(0.30–21.37)	0.390
About 40–60%	0.91	(0.14–5.80)	0.921	1.28	(0.18–9.39)	0.807
About 80%	0.78	(0.12–5.18)	0.795	0.93	(0.12–7.09)	0.944
Almost all	0.58	(0.09–3.53)	0.552	0.78	(0.11–5.41)	0.798

Multiple logistic regression analysis adjusted for the variables in this table. CI: Confidence interval.

## Data Availability

The datasets used and/or analyzed during the current study are available from the corresponding author on reasonable request.
